# Draft Genome Sequence of *Lactococcus lactis* subsp. *cremoris* HP^T^, the First Defined-Strain Dairy Starter Culture Bacterium

**DOI:** 10.1128/genomeA.00107-14

**Published:** 2014-03-06

**Authors:** Suzanne C. Lambie, Eric Altermann, Sinead C. Leahy, William J. Kelly

**Affiliations:** AgResearch, Ltd., Grasslands Research Centre, Palmerston North, New Zealand

## Abstract

*Lactococcus lactis* subsp. *cremoris* HP^T^ has been widely used in studies of the metabolism of lactococcal dairy starter cultures. A comparison of the draft HP^T^ genome with those from other strains of *L. lactis* subsp. *cremoris* will aid our understanding of the domestication and evolution of these important industrial cultures.

## GENOME ANNOUNCEMENT

*Lactococcus lactis* subsp. *cremoris* HP (NCDO 607, ATCC 19257, DSM 20069, formerly *Streptococcus cremoris*) is the type strain of *L. lactis* subsp. *cremoris* (1) and the first defined-strain dairy starter culture bacterium used for cheddar cheese production. It was isolated from a mixed-strain starter in 1935 and used in most of the early observations on bacteriophages as a cause of starter culture failure (2). The HP designation originates from the Hopelands and Papatawa dairy factories in New Zealand, where it was first isolated (3). Subsequently, HP^T^ has been used in many studies focused on lactococcal dairy starters, particularly the characterization of the cell envelope proteinase (4), although its tendency to produce bitter-flavored cheese means that other strains are now favored for industrial use (5). HP^T^ is unable to deaminate arginine and fails to grow in 4% NaCl or at 40°C, giving it the subspecies *cremoris* phenotype (1). Its carbohydrate utilization profile is much more limited than that of wild-type *L. lactis* subsp. *cremoris* strains (6), with acid only being produced from *N*-acetyl glucosamine, cellobiose, fructose, galactose, glucose, lactose, and mannose. HP^T^ harbors several plasmids, and the one encoding the cell envelope proteinase (pHP003, 13.4 kb) has been sequenced (5). The HP^T^ chromosome has undergone rearrangement so that the positions of the rRNA operons differ from those found in most *L. lactis* strains (7).

The draft genome sequence of HP^T^ was determined using pyrosequencing of 3-kb mate paired-end sequence libraries on a 454 GS FLX platform with Titanium chemistry (Macrogen, South Korea). Pyrosequencing reads were assembled using the Newbler assembler version 2.5.3 (Roche 454 Life Sciences, USA), resulting in 213 contigs, the largest of which is 104,897 bp. Protein-coding genes were identified by Glimmer (8), and a GAMOLA/ARTEMIS software suite (9, 10) was used to manage genome annotation. The assignment of protein functions to open reading frames (ORFs) was performed manually using results from BLASTp and the COG (Clusters of Orthologous Groups), Pfam, and TIGRFAM databases (11–13).

The draft genome sequence of *L. lactis* subsp. *cremoris* HP^T^ is 2,276,325 bp, with a G+C content of 36.7% and 2,374 predicted coding sequences. The gene content is very similar to those of other *L. lactis* subsp. *cremoris* dairy starter strains, but the genes for starch/maltose breakdown, tryptophan metabolism, and tolerance to osmotic stress are missing, as has been reported previously (14). It is likely that the high contig number results from the presence of numerous transposases that are characteristic of these dairy starter strains. The genome also contains ~120 ORFs that match various lactococcal phages.

A comparison of the HP^T^ genome with those from three other industrially used *L. lactis* subsp. *cremoris* strains, A76, SK11, and UC509.9 (15–17), shows a clear relationship between the strains and will help in understanding the origins, domestication, and evolution of these important industrially used dairy starter bacteria.

### Nucleotide sequence accession numbers.

This whole-genome shotgun project has been deposited at DDBJ/EMBL/GenBank under the accession no. JAUH00000000. The version described in this paper is version JAUH01000000.
